# Little Utility of Fungal Blood Cultures in Surgical and Burn Intensive Care Units

**DOI:** 10.1128/spectrum.00228-22

**Published:** 2022-06-28

**Authors:** Jacqueline Babb, Audra Clark, Donna Gaffney, Kareem Abdelfattah, Bonnie C. Prokesch

**Affiliations:** a Department of Surgery, UT Southwestern Medical Center, Dallas, Texas, USA; b Parkland Hospital, Dallas, Texas, USA; c Department of Internal Medicine, UT Southwestern Medical Center, Dallas, Texas, USA; University of Mississippi Medical Center

**Keywords:** fungemia, candidemia, ICU, blood culture, critical care

## Abstract

Critically ill patients are at risk for fungal infections, but there is a paucity of data regarding the clinical utility of dedicated fungal blood cultures to detect such infections. A retrospective review was conducted of patients admitted to the surgical and burn intensive care units at Parkland Memorial Hospital between 1 January 2013 and 31 December 2017 for whom blood cultures (aerobic, anaerobic, and/or fungal cultures) were sent. A total of 1,094 aerobic and anaerobic blood culture sets and 523 fungal blood cultures were sent. Of the aerobic and anaerobic culture sets, 42/1,094 (3.8%) were positive for fungal growth. All fungal species cultured were *Candida.* Of the fungal blood cultures, 4/523 (0.76%) were positive for growth. Fungal species isolated included Candida albicans, Aspergillus fumigatus, and Histoplasma capsulatum. All 4 patients with positive fungal blood cultures were on empirical antifungal therapy prior to results, and the antifungal regimen was changed for 1 patient based on culture data. The average duration to final fungal culture result was 46 days, while the time to preliminary results varied dramatically. Two of the four patients died prior to fungal culture results, thereby rendering the culture data inconsequential in patient care decisions. This study demonstrates that regular aerobic and anaerobic blood cultures sets are sufficient in detecting the most common causes of fungemia and that results from fungal cultures rarely impact treatment management decisions in patients in surgical and burn intensive care units. There is little clinical utility to routine fungal cultures in this patient population.

**IMPORTANCE** This study demonstrates that regular aerobic and anaerobic blood culture sets are sufficient in detecting the most common causes of fungemia, and thus, sending fungal blood cultures for patients in surgical and burn intensive care units is not a good use of resources.

## INTRODUCTION

Fungemia is a common infection among patients in intensive care units, accounting for over 10% of bloodstream infections, with the majority composed of candidemia or in relation to invasive candidiasis (1–5). Critically ill patients have many risk factors predisposing them to the development of fungal infections in general, including but not limited to the high likelihood of experiencing multiple invasive interventions, the presence of indwelling catheters (namely, central lines), immunocompromised status, exposure to broad-spectrum antibiotics, and multiorgan dysfunction (5–9). Burn patients have an especially high risk of fungal infections given the skin disruption that occurs secondary to burn injury (10, 11). Fungal infections in burn patients are associated with a high rate of mortality, and invasive fungal invasion of burn wounds shown on autopsies has been reported in 28 to 44% of patients (10, 12).

Although fungemia is frequently seen in this cohort of patients, little evidence exists in the literature to help distinguish patients at increased risk for developing fungal bloodstream infection, and diagnosis can be difficult (5, 7–9). Positive blood cultures have historically been the gold standard for the diagnosis of fungemia (8, 9). Fungemia can be diagnosed with fungal isolator blood culture bottles, in addition to traditional blood culture sets, which include an aerobic bottle and an anaerobic blood bottle per each set of cultures (13). At the advent of fungal isolator cultures, which involves lysis centrifugation technology, early studies showed enhanced recovery of fungal pathogens compared to traditional blood cultures. However, as fungal cultures evolved, traditional aerobic and anaerobic blood culture sets became more sensitive as well, leading to an increased growth of fungal organisms on aerobic and anaerobic blood cultures (13). Currently, the majority of modern traditional aerobic and anaerobic blood culture sets allow for growth of the most common fungal pathogens in critical care patients, including *Candida* and Cryptococcus species, without special preparations or additional steps in the microbiology laboratory (14, 15). Fungal cultures facilitate the growth of rare and endemic fungal species such as *Blastomycosis*, *Histoplasmosis*, and *Coccidiosis*, as well as molds such as Aspergillus species, as these pathogens require extended incubation times and specialized media for growth (13). Fungal infections in critically ill burn patients usually involve *Candida* and Aspergillus, and Aspergillus appears to be more common in patients with fungus-associated death (10). While medical therapy is readily available for fungal infections, treatment involves risks such as medication toxicities (including hepatotoxicity and nephrotoxicity), drug interactions, resistance, and high cost (13, 14).

While the Infectious Diseases Society of America (IDSA) has guidelines regarding treatment and management of candidemia, they do not discuss diagnosis (14). Moreover, while the European Organization for Research and Treatment of Cancer and the Mycoses Study Group Education and Research Consortium (EORTC/MSGERC) discusses definitions and diagnoses of invasive fungal diseases, the group acknowledges that the definitions may not be applicable to patients in the intensive care unit, further revealing that there is a true paucity of data regarding fungal disease in this patient population (5). Especially in critically ill patients, it can be difficult to confirm fungemia, often leading providers to rely on clinical judgment with regard to risk factors and clinical picture, resulting in prophylactic or empirical therapies (5–7). In fact, this approach has been favored as opposed to relying on objective data (such as actual positive fungal cultures) to confirm diagnosis, as it is well known that fungemia is associated with high mortality and morbidity and early therapy is clearly beneficial (16–18). Additionally, blood cultures can be falsely negative, especially in the setting of transient fungemia, and often require over 48 to 72 h to grow (7–9, 14). This combination of factors can result in both misdiagnosis and underdiagnosis of a potentially fatal infection. We sought to better understand the true utility of fungal blood cultures in patients in the surgical intensive care unit (SICU) and burn intensive care unit (BICU) settings.

We hypothesized that in this cohort of patients, routine blood culture sets, defined as aerobic and anaerobic blood cultures, were effective and efficient in the identification of candidemia and that fungal blood cultures rarely impacted clinical decision making regarding antifungal therapy, and thus there is little utility in routine use of fungal blood cultures in the SICU and BICU settings.

## RESULTS

**FIG 1:**
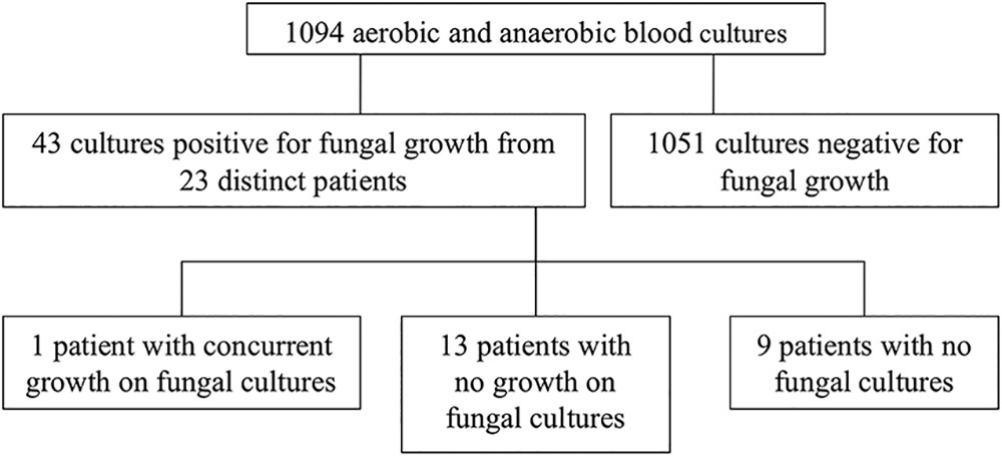
Positive fungal culture results from aerobic and anaerobic blood cultures.

**FIG 2:**
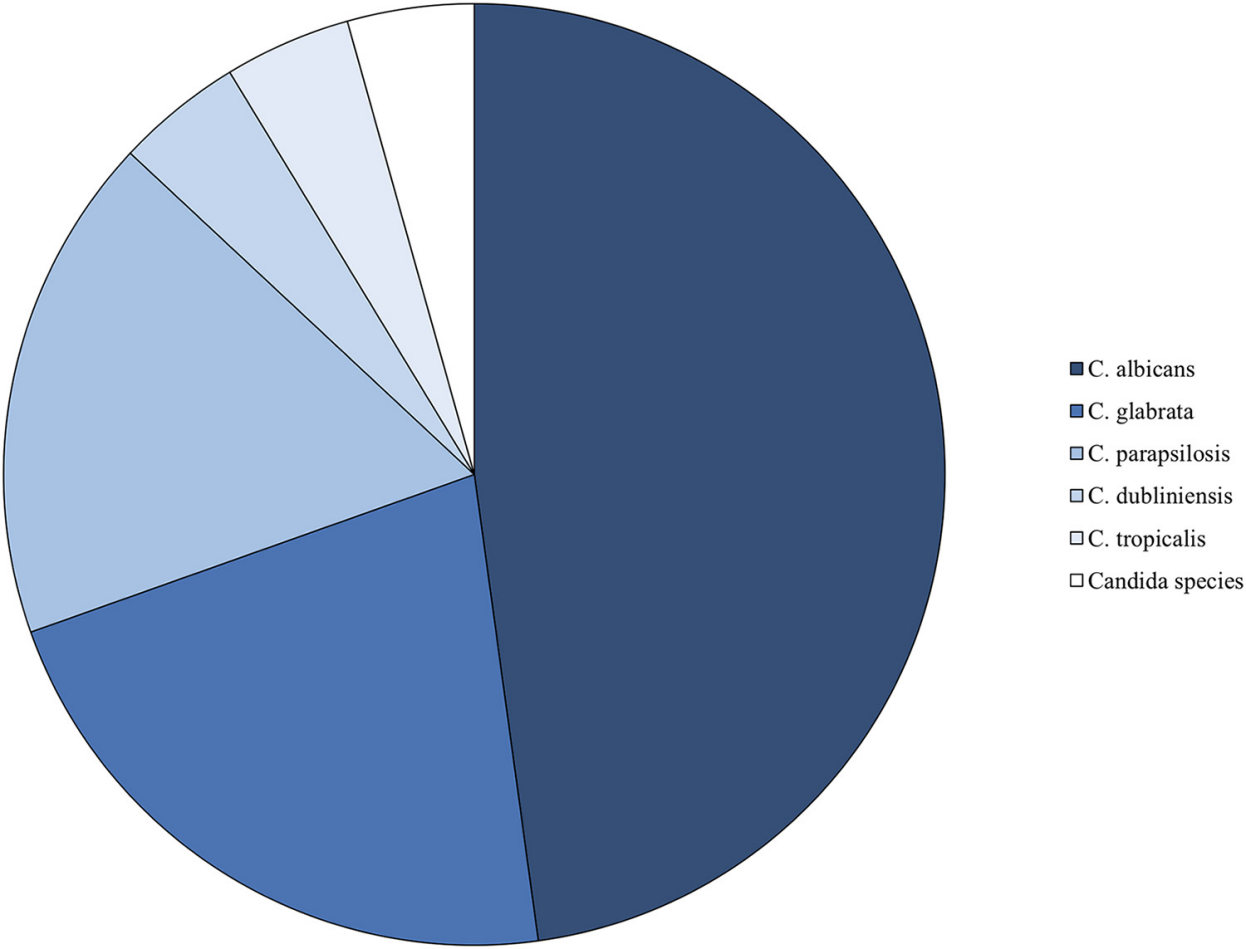
Fungal species isolated on aerobic and anaerobic blood cultures.

A total of 1,094 aerobic and anaerobic blood culture sets and 523 fungal blood cultures were drawn between 1 January 2013 and 31 December 2017 from patients in the SICU and BICU. Of the aerobic and anaerobic culture sets, 42/1,094 (3.8%) were positive for fungal growth within 5 days after collection. These 42 cultures were from 23 distinct patients, of which 1 had growth on fungal blood cultures, 13 had no growth on fungal blood cultures, and 9 did not have fungal cultures drawn (Fig. 1). Of the 23 patients with positive fungal growth on routine culture sets, 9 patients (39%) were in the SICU and 14 patients (61%) were in the BICU. Fungal species cultured on routine blood culture sets included Candida albicans, Candida glabrata, Candida dubliniensis, Candida tropicalis, and Candida parapsilosis (Fig. 2). Of the fungal blood cultures, 4/523 (0.76%) were positive for fungal growth. Fungal species that were cultured on fungal blood culture included Candida albicans, Aspergillus fumigatus, and Histoplasma capsulatum (Table 1).

**TABLE 1:** Demographic and clinical characteristics of patients with positive fungal isolator blood cultures^*a*^

Characteristic	Data for patient no. (location):			
	1 (BICU)	2 (BICU)	3 (BICU)	4 (BICU)
Age (yrs)	38	38	43	52
Gender	M	F	M	M
Aerobic and anaerobic culture isolate results	No growth	Yeast	Pseudomonas	No growth
Fungal culture isolate results	Candida albicans	Aspergillus fumigatus	Candida albicans	Histoplasma capsulatum
Diagnosis	65% TBSA, car fire	85% TBSA, house fire	80% TBSA, self-inflicted	44% TBSA
Treatment before results	Voriconazole	Micafungin to amphotericin	Voriconazole	Micafungin to amphotericin to voriconazole
Treatment after results	None—completed 10 days’ course of voriconazole	None—death prior to positive fungal culture	Amphotericin	None—death prior to positive fungal culture
Immunocompromised	No	No	No	No
Indication for culture	Sepsis, increasing pressor requirements	Sepsis, wound cultures with Aspergillus flavus and A. fumigatus	Sepsis, ARDS vs pneumonia, leukocytosis	Sepsis, increasing WBC, temp, and pressors Wound cultures with *Fusarium*
Indwelling line	Central line × 2	Central line	Central line	Central line × 2
Patient outcome	Alive	Death	Alive	Death
Fungal blood culture report history (days until preliminary report)	*Candida* (17)	Fungus (7) Aspergillus sp. (10) A. flavus (15)	Yeast (5) C. albicans (6)	Fungus (43) *Histoplasma* (53)
Days to final report for fungal culture	47	34	49	53
Days to final report of aerobic and anaerobic blood culture	NA	Yeast—3	NA	NA
Additional information	1/3 fungal cultures positive, 4 routine blood cultures negative		Pseudomonas in blood and respiratory cultures, yeast in urine	

a ARDS, acute respiratory distress syndrome; BICU, burn intensive care unit; TBSA, total body surface area; WBC, white blood cell.

Further review of the 4 patients with positive fungal blood cultures was pursued, and details regarding their overall clinical conditions, the results of concurrent traditional blood culture sets, timing to positivity of the fungal blood cultures, and the effects of fungal culture results on clinical management can be found in Table 1. All 4 patients were in the BICU and had fungal cultures drawn in the setting of septic shock. Two out of the four patients also had tissue cultures positive for fungal growth, which resulted as positive prior to the blood culture results. All 4 patients were on empirical antifungal therapy prior to the reporting of fungal blood culture data, and the antifungal regimen was changed for only 1 patient following fungal culture results. In this study, fungal isolates took various times to result, with ranges for preliminary results of from 5 to 43 days, with an average time to finalized reporting of 46 days (Table 1). Two of the patients died prior to the reporting of final fungal culture results.

## DISCUSSION

Fungemia is one of the most common types of bloodstream infections in the intensive care unit, and critically ill patients have many risk factors that make them more susceptible to fungal infection (5). There are currently no guidelines or comprehensive protocols from professional societies (including the IDSA) regarding details of diagnosis of fungal bloodstream infections, especially in critically ill patients (5, 14). Historically, dedicated fungal cultures were determined to be the gold standard for the diagnosis of fungemia. However, the EORTC/MSGERC recommendations for invasive fungal diseases do not even mention the use of fungal cultures for diagnosis of invasive candidiasis. Rather, the group suggests collecting two sets of aerobic and anaerobic blood cultures prior to initiation of antifungal therapy (5). Our data corroborate that fungal cultures are overutilized in the SICU and BICU settings, aligning with data from prior studies in other patient populations finding that fungal blood cultures are often ordered unnecessarily with little benefit to patient care and outcome and with increased adverse effects such as increased cost, unnecessary treatment, and harmful reactions to such treatment (13, 19).

Despite the fact that many fungal cultures were drawn in the critically ill patients included in this study, very few (only 4 out of 523) were positive, and most importantly, positive fungal growth was more commonly seen on routine aerobic and anaerobic cultures than on fungal cultures. Interestingly, more than 50% of the patients who had fungal growth on routine blood culture sets did not have growth on dedicated fungal cultures. Because routine blood culture sets allow for the growth of the most common fungal organisms causing fungemia in this patient population (including *Candida* and Cryptococcus), it is not surprising that there were many more cases of candidemia diagnosed via regular blood cultures than via fungal cultures. In fact, *Candida* species have been shown to grow better and faster on modern routine aerobic and anaerobic blood cultures than on dedicated fungal blood cultures (13). Although positive fungal blood cultures have historically been the gold standard for the diagnosis of fungemia, this study indicates that routine blood culture sets are sufficient and that a dedicated fungal culture is not usually necessary to diagnose the most common causes of fungemia in patients in the SICU and BICU.

Previous studies of patients in the medical intensive care unit have shown that standard cultures sets are often sufficient compared with dedicated fungal cultures for diagnosing fungemia, which our study corroborates in the critically ill surgical patient cohort (5, 13, 19). In addition, fungal cultures have been known to take substantially longer to result, with previous data suggesting 48 to 72 h at a minimum (7–9, 14). As mentioned above, in this study, fungal cultures took various times to result, with an average time to finalized reporting of 46 days. Most importantly, we found that in this patient cohort fungal cultures did not impact clinical management even when they were found to be positive. Clinicians usually guide management of suspected fungemia based on clinical judgment rather than culture data because fungemia is difficult to diagnose, at-risk patients are difficult to identify, and the risks of not detecting fungemia are extremely high, and at times, fatal (5–9, 16). Moreover, the long duration to obtain final results on fungal cultures reduces the utility of fungal blood cultures. Because fungemia is known to have a high association with morbidity and mortality, a delay in diagnosis while awaiting fungal culture results is unacceptable (16–18). In fact, results of fungal cultures can even take longer than the typical treatment duration of fungemia and may not even result before the patient is already deceased due to complications from the infection, with both findings seen in our cohort. For the above-described reasons, many clinicians guide management with both prophylactic and empirical therapy based on clinical judgment (6, 7, 16). Additionally, often even with culture data present, management does not change. Our study suggests that fungal cultures have very little impact on clinical management, as most cultures were unrevealing, and in the 4 patients with positive cultures from fungal isolators, therapy was only changed in one instance following fungal culture results. In this instance, the change made was, in fact, not needed, as the antifungal agent that was already being utilized offered sufficient coverage for the organism that grew on culture. The patient had empirically been on voriconazole, and once Candida albicans was isolated, therapy was unnecessarily escalated to amphotericin (Table 1). Thus, while culture data in this case did result in a change in clinical management, the change was not necessary and, in fact, could have potentially resulted in more harm (due to the many toxicities related to amphotericin) than good.

As such, although fungal blood cultures themselves pose little to no risk to the patient, there are inherent adverse effects and risks to the patient when unnecessary diagnostic studies are ordered. While this study was not specifically designed to assess the impact of superfluous diagnostic testing on health care costs, the overall cost of work up of an additional blood culture (i.e., fungal blood cultures in addition to routine blood cultures) can be of significance to the institution. Blood culture costs for aerobic and anaerobic culture bottle sets at our institution are $6.06, and fungal isolate culture bottles are $7.77. Although this seems relatively insignificant in the scope of critical care medicine, the price listed does not account for the additional costs of supplies and culture media, laboratory time, processing and storing, and the personnel involved in all steps from obtaining cultures to maintaining and interpreting them. Thus, the overall cost of work up of an additional blood culture (i.e., fungal blood cultures in addition to routine aerobic and anaerobic blood cultures) can be of significance to the institution. Previous studies have demonstrated that a more judicious use of fungal cultures in one department saved more than $300,000 in 1 year (13).

Although our study has multiple strengths, including a large number of cultures, a vast patient cohort, the setting of a level 1 trauma center affiliated with a large academic institution, and both a SICU and a BICU population, there are several limitations. First, the patient cohort consists mostly of young trauma patients who were immunocompetent prior to admission. The study’s intensive care units do not routinely admit patients with immunocompromised conditions, and extremely few transplant patients are admitted to these units. In addition, although a large number of fungal cultures were drawn in this group of patients, not all patients had both fungal isolate cultures and standard blood culture sets drawn simultaneously, thus preventing direct comparison in every case. Furthermore, the circumstances surrounding negative fungal blood cultures were not analyzed in depth, and thus, whether or not the negative results of the fungal isolator cultures impacted clinical management was not assessed. Further studies will need to be done to determine the utility of fungal blood cultures in different patient populations and the impact of both positive and negative blood cultures on clinical management.

### Conclusions.

Although fungemia is one of the most common infections in SICU and BICU patients and, historically, fungal cultures have been utilized to diagnose and guide management of fungal infections, there is little clinical utility to routine fungal cultures presently, as regular aerobic and anaerobic blood cultures are sufficient to detect the most common forms of fungemia. The limited number of positive fungal cultures in this patient cohort indicates that fungal cultures are not necessary to diagnose candidemia and, more importantly, that routine aerobic and anaerobic cultures are more sensitive and specific in the diagnosis of candidemia than fungal cultures. Furthermore, results from fungal cultures may not impact clinical management and have potential adverse effects such as increased cost and potential risks associated with inappropriate antifungal therapy. As such, fungal isolator cultures should only be ordered in very limited clinical settings in the BICU and SICU patient populations.

## MATERIALS AND METHODS

We conducted a single-institution retrospective study at Parkland Memorial Hospital, an 862-bed, level 1 trauma center, American Burn Association-verified burn center, and safety-net hospital for Dallas County serving as the primary teaching site for the University of Texas Southwestern Medical School. Institutional review board approval was obtained. All patients admitted to the SICU and BICU between 1 January 2013 and 31 December 2017 in which blood cultures (aerobic, anaerobic, and/or fungal cultures) were sent were included in this study. Patients were identified by their location in the hospital during time of admission, and culture data were collected from the microbiology laboratory electronic database. Blood specimens are collected in Wampole Isolator tubes and processed in the standard fashion within 16 h of collection. The isolator sediment was then inoculated onto a Sabouraud dextrose with brain heart infusion agar (SABHI) slant, potato dextrose agar slant, and brain heart infusion agar (BHI) with a 5% sheep blood slant and incubated. These are examined by technicians weekly for 6 weeks to detect fungal growth.

The electronic medical record was further queried to assess relevant demographic details including age, gender, diagnosis, antifungal therapies, immunocompromised status, indication for fungal blood culture when sent, and time to positive culture in the patients within the cohort with positive fungal blood cultures. Results were reported in a descriptive manner without need for statistical analysis due to the small nature of the study.
